# The effects of graft source and orientation on outcomes after ablation of a branched peripheral nerve

**DOI:** 10.3389/fncel.2022.1055490

**Published:** 2022-11-14

**Authors:** JuliAnne E. Allgood, Kelly C. Santos Roballo, Bridger B. Sparks, Jared S. Bushman

**Affiliations:** Division of Pharmaceutical Sciences, University of Wyoming, Laramie, WY, United States

**Keywords:** branched peripheral nerve injury, nerve allograft, immunogenicity, functional outcomes, retrograde labeling

## Abstract

Segmental peripheral nerve injuries (PNI) are the most common cause of enduring nervous system dysfunction. The peripheral nervous system (PNS) has an extensive and highly branching organization. While much is known about the factors that affect regeneration through sharp bisections and linear ablations of peripheral nerves, very little has been investigated or documented about PNIs that ablate branch points. Such injuries present additional complexity compared to linear segmental defects. This study compared outcomes following ablation of a branch point with branched grafts, specifically examining how graft source and orientation of the branched graft contributed to regeneration. The model system was Lewis rats that underwent a 2.5 cm ablation that started in the sciatic nerve trunk and included the peroneal/tibial branch point. Rats received grafts that were rat sciatic autograft, inbred sciatic allograft, and inbred femoral allograft, each of which was a branched graft of 2.5 cm. Allografts were obtained from Lewis rats, which is an inbred strain. Both branches of the sciatic grafts were mixed motor and sensory while the femoral grafts were smaller in diameter than sciatic grafts and one branch of the femoral graft is sensory and the other motor. All branched grafts were sutured into the defect in two orientations dictated by which branch in the graft was sutured to the tibial vs peroneal stumps in recipients. Outcome measures include compound muscle action potentials (CMAPs) and CatWalk gait analysis throughout the recovery period, with toluidine blue for intrinsic nerve morphometry and retrograde labeling conducted at the 36-week experimental end point. Results indicate that graft source and orientation does play a significant role earlier in the regenerative process but by 36 weeks all groups showed very similar indications of regeneration across multiple outcomes.

## Introduction

Damage to the peripheral nervous system (PNS) is common, with hundreds of thousands of new peripheral nerve injuries (PNIs) occurring in the United States (PNI) each year (Liu et al., 2012; Catala and Kubis, 2013). The most common causes of PNIs are motor vehicle accidents or violent trauma (Pan et al., 2020). Recovery is often slow and incomplete for even simple lacerations (Wang et al., 2019). Outcomes are worse for injuries that ablate sections of the nerves (segmental ablations) and if the PNI is long distances from the innervation target tissues (Cinteza et al., 2015). Permanent disability is common.

One of the reasons that PNIs greater distances from innervation tissues have poorer outcomes is the increasing number of branch points likely to be distal to the injury; there are approximately 80 km of peripheral nerves throughout a human body in an extensively branching organization (Catala and Kubis, 2013). When regenerating axons reach a branch point, the leading axons can sample pathways and appear to initially regenerate randomly into either branch (Madison et al., 1996; Brushart et al., 1998; Witzel et al., 2005). This dilutes the regenerative effect due to pruning and functional irrelevance of motor neurons that misroute into sensory branches and increases the likelihood that axons do not regenerate to the same target tissue they innervated prior to injury (Brushart, 1993; Witzel et al., 2005). Plasticity of the central and peripheral nervous systems to compensate for innervation changes is considerable, but is not unlimited (Navarro, 2009).

Unlike linear segmental PNIs that have been extensively studied and addressed with a variety of highly compared techniques and technologies (i.e., autografts, acellular nerve allografts (ANAs), conduits/wraps etc.) (Jones et al., 2016; Safa and Buncke, 2016), very little has been documented for PNIs that ablate a branch point. The process of regeneration at branch points has primarily been investigated in the context of what occurs following segmental or sharp laceration PNIs proximal to branch points, primarily with the rodent femoral nerve model (Irintchev, 2011). The posterior division of the femoral nerve divides into a sensory saphenous branch and a motor branch that innervates the quadriceps muscle, making this a useful model because retrograde labeling can be used to quantify misrouting of regenerating axons (Irintchev, 2011).

Data from these proximal PNIs to the femoral revealed the process of preferential motor reinnervation; where motor neurons are more likely to regenerate into the motor branch and avoid the sensory branch (Brushart et al., 1998). Preferential motor reinnervation has been attributed to biological cues both within the motor branch as well as cues initiated when the first motor neurons to innervate a distal muscle trigger a process that attracts more slowly regenerating motor neurons down the same path (Brushart, 1988; Martini et al., 1992). This process reduces the number of motor neurons that regenerate into the sensory branch in the femoral nerve after injury (Madison et al., 2007). It has been recently observed that sensory axons inhibit extension of motor neuron axons in 3-dimensional organotypic cultures *in vitro* (Brushart et al., 2020). If this effect is confirmed *in vivo*, it would further support that regeneration of motor neurons at sensorimotor branch points, such as the posterior femoral nerve, is dependent upon both positive and negative cues.

Despite these seminal studies showing the importance of pathfinding of regenerating axons at branch points and the known branching complexity of the PNS, studies on ablated branch points are lacking. Anatomically, ablated branch points present additional complexity simply because there is an increased number of nerve stumps to surgically manage. The lack of comparatively validated methods to manage ablated branch points creates additional uncertainty in the clinical management of these injuries. For example, the attempting to direct regeneration across a branched ablation creates the risk to misdirect a disproportionate number of axons into any of the distal branches. This would potentially leave other branches with too few regenerating axons to mediate function. Disproportionate axonal regeneration at branch points is also a possibility for PNIs to linear segments proximal to branch points, but this would not be a controllable variable in such cases.

We have previously investigated branched ablation PNIs from the perspective that a live nerve graft that matches the anatomy of the defect site would likely be a successful bridging material. Because autografts would not be commonly available for ablated branch points, we investigated the use of live (not decellularized) allografts and developed methods of localized immune suppression to avoid the need for systemic immune suppression (Santos Roballo et al., 2019). Immunosuppression is necessary with mismatched donors and recipients to prevent rejection of the nerve allografts (Siemionow and Sonmez, 2007; Roballo et al., 2022). The model in these previous studies was a 2.0 branched ablation of the sciatic nerve in Lewis rats that included the peroneal/tibial branch point. This defect that bridged with live allografts obtained from major histocompatibility mismatched Sprague Dawley donors (Hurt et al., 2004). 2.0 cm branched autografts, consisting of the same segment cut out and re-sutured into the defect in Lewis rats, were conducted as a control group. Data indicated robust regeneration occurred for autografts and allografts with localized immune suppression into both the tibial and peroneal branches.

Successful regeneration down both branches in this branched ablation PNI model allows for the investigation of more fundamental concepts explored in this report. Specifically, experiments sought to determine how graft source and orientation would affect the regenerative process after a branched ablation PNI of the sciatic nerve. The experimental model is a 2.5 cm ablation of the sciatic nerve that included the tibial and peroneal branch point in rats. A 2.5 cm sciatic or femoral branched graft was sutured into the defect in two orientations; one where the tibial and motor branches of the sciatic and femoral grafts, respectively, were sutured to the tibial stump in the defect and the peroneal and saphenous branches of the grafts, respectively, sutured to the peroneal stump. The other was a switched orientation where the tibial and motor branches of the sciatic and femoral grafts were respectively sutured to the peroneal stumps and the peroneal and saphenous branches sutured to the tibial stumps. Experiments were conducted with inbred Lewis donors and recipient rats to avoid the need for immune suppression and this potential source of variability (Avramut and Achim, 2003).

Experiments were carried out to 36 weeks and outcome measures included compound muscle action potentials (CMAPs) to distal muscle innervation targets of the tibial and peroneal nerves, CatWalk gait analysis, nerve morphometry from toluidine blue sections of each graft branch and retrograde labeling. Results indicated that the graft source and orientation initially caused some significant differences in CMAPs and CatWalk but that these differences became less evident by the study end point of 36 weeks. Nerve morphometry suggested axon regeneration was robust down both branched with all grafts, with some differences and trends according to graft type and orientation. Retrograde labeling showed that motor axons were more abundant than sensory axons in all groups but switching the orientation of the branches increased the number of sensory axons in the distal peroneal branch compared to the distal tibial branch. Together, this data suggests that regeneration following branched ablation PNI of the sciatic nerve can be reliably achieved with grafts that differ in their size and sensorimotor complexity.

## Materials and methods

### Animal acquisition and care

Animals for this study were acquired, cared for, and used in accordance with the NIH Guide for the Care and Use of Laboratory Animals under protocols approved by the University of Wyoming IACUC. Rats were housed at ambient temperature with stable humidity on a 12-h day-night cycle and free access to food and water. Lewis and Sprague-Dawley (SD) rats were obtained from Charles River Laboratories, Wilmington, MA, USA. Animals were housed individually from the beginning of CatWalk training. A total of 39 Lewis rats were randomly assigned to one of six surgical groups (*n* = 6) or an uninjured control group (*n* = 3). One rat in the sciatic autograft group developed an ovarian tumor that hindered its ability to walk and was sacrificed at 28 weeks post operatively (PO). Data for this rat was removed from analysis, leaving the Lewis sciatic autograft group with *n* = 5 animals. A total of 12 SD rats were randomly assigned to the sciatic autograft group (*n* = 6) or the sciatic allograft group (*n* = 6). Two rats from the SD sciatic allograft group had unrepairable autophagy and were sacrificed 1 week PO. Data for these rats was removed from analysis leaving the SD sciatic allograft group with *n* = 4 animals.

### Harvest and storage of allografts

Male Lewis rats were used as donors to obtain inbred allografts in Lewis-to-Lewis. Male Sprague Dawley rats were used as donors in the outbred Sprague Dawley to Sprague Dawley experiments. Rats were sacrificed by isoflurane overdose followed by cervical dislocation. After euthanasia, both the right and left sciatic and femoral nerves were harvested, carefully stripped of connective tissue, and stored in 1× PBS on ice for between 1 and 5 h prior to implantation.

### Surgical procedure and post-operative care

Surgery and post-operative care was as previously described (Santos Roballo et al., 2019). Briefly, female Lewis rats weighing an average of 219 ± 13 g were anesthetized with 2% inhaled isoflurane, their left hind limb shaved, and the incision site sterilized with 3× wipes with betadine (VWR, BDH7207-4) and 100% isopropyl alcohol (Sam’s West, 645081). Vaseline was placed over the eyes to prevent drying, and rats were administered 2 mg/kg bupivacaine (VetOne, 510212) subcutaneously at the incision site and 5 mg/kg Baytril (Bayer, 84744158) and 0.03 mg/kg buprenorphine (PAR pharmaceuticals, 42023-179-05) subcutaneously in the opposite hind limb. Rats were placed on a 38°C circulating water temperature-controlled surface, with toe pinch used to confirm sufficient anesthesia, and the surgical site draped with sterile pads. Using sterile no-touch technique, a ∼3 cm incision was made to the skin above the femur and the fascia between the gluteus superficialis and biceps femoris were gently separated to expose the sciatic nerve. A 2.5 cm section of the sciatic nerve was removed beginning ∼1 mm distal to the external obturator tendon and extending distally, which was 1 cm or more past the peroneal-tibial branch point. Grafts were trimmed to 2.5 cm and implanted with 9-0 sutures (esutures, AA-2628). Musculature and skin were closed with 6-0 sutures (esutures, A697N) and animals were allowed to recover on the heated surface before being placed into individual cages. Experiments with Sprague Dawley rats used the same procedure, but had 2.0 cm defects and grafts implanted instead of 2.5 cm.

For post-operative care, rats received 0.03 mg/kg buprenorphine twice daily subcutaneously for 3 days after surgery and one dose of 5 mg/kg Baytril subcutaneously for 7 days after surgery. Animals were monitored at least twice per day for signs of distress or autophagy for the first 7 days and at least once per day thereafter. Six of the recipient Lewis rats were found to have disrupted the sutures in their skin within 4 days of surgery but without any tissue autophagy or autotomy. After resuturing when required, these rats were fitted with Elizabethan collars (Kent Scientific, EC404VS) until 10 days after surgery and remained in the study.

### Gait analysis

The Noldus Catwalk XT system was used to evaluate behavioral recovery following sciatic nerve injury as we previously described (Osimanjiang et al., 2022). Briefly, animals were placed on one end of an open-ended tunnel with overhead red-light illumination and green light walkway illumination (0.12 intensity). Animals were acclimated and conditioned to the system for four weeks prior to surgery. Baseline data was collected when animals voluntarily ran three times across the platform in compliance with the set parameters, 60% or less speed variation and less than 15 s duration, while a camera fixed at 42 cm below the platform recorded foot placement. Lewis rats ran the CatWalk for data collection every two weeks for 36 weeks with each animal requiring a minimum of three compliant runs per trial. Data was auto classified by the CatWalk software and a follow-up performed by individuals blinded to experimental groups to correct any misidentified steps. The cadence in steps/second, max contact mean intensity, and right front (RF)/Left Hind (LH) coupling were calculated using the CatWalk software after classification was complete. Additionally, toe spread, print length, and intermediate toe spread were also manually measured according to previously published protocols for all left hind feet to allow for the CatWalk software to calculate sciatic functional index (SFI) (De Medinaceli et al., 1984; Bozkurt et al., 2011; Isvoranu et al., 2021).

### Electrophysiology

Recovery down each sciatic nerve branch was assessed with electrophysiological recordings from needle electrodes in the foot muscles innervated by the tibial and peroneal branches as previously described (Werdin et al., 2009; Roballo and Bushman, 2019; Santos Roballo et al., 2019). Using the Viking NCS EMG EP IOM System, Compound Muscle Action Potentials (CMAPs) were collected using five electrodes (Natus, 019-476600): ground, reference, recording, and an anode/cathode pair for stimulating the muscle. Placement of the ground and reference electrodes were subcutaneously placed on the lateral side of the 5th metatarsal running dorsal through the heal, and on the lateral side of the 5th metatarsal running to the anterior, respectively. Subcutaneously the recording electrode was inserted on the dorsal foot muscle between the 2nd and 3rd metatarsals for peroneal branch stimulation and on the plantar muscle between the 2nd and 3rd metatarsal for stimulation of the tibial branch. Stimulation using the anode/cathode pair was done percutaneously at the ankle in the space between the tibia and calcaneal tendon. The top three highest amplitudes and top three lowest latencies were averaged and used for analysis at each time point for each animal. CMAPs recordings were taken prior to surgery and every 4 weeks post operatively (PO) for the duration of the 36-week study.

### Retrograde labeling

Two days prior to euthanasia, animals were anesthetized with 2% isoflurane, the nerve exposed, and microinjection syringes fitted with Nanofil 36 G needles (World Precision Instruments, NF36BV-2) inserted under the epineurium. Cholera Toxin subunit B (CTb) was slowly injected into each branch. The tibial branch was injected with 4 μl CTb conjugated with Alexa Fluor 488 (ThermoFisher, C22841) diluted 1:100 in 1× PBS (ThermoFisher, 14190235) and the peroneal branch was injected with 2–3 μl CTb conjugated with Alexa Fluor 555 (ThermoFisher, C22843) diluted 1:100 in 1× PBS. Needles were left inserted into nerves for 60 s following injection. The muscle and skin were then sutured using 6-0 sutures, and the animals were allowed to recover on the heated pad. Animals were also administered 0.03 mg/kg buprenorphine twice daily until sacrifice.

### Euthanasia and tissue collection

At the end point, animals were euthanized via perfusion with 4% paraformaldehyde (PFA) in 1× PBS (Cat. No. 14200075, Life Technologies). Briefly, animals were anesthetized via isoflurane, the chest cavity opened, and animals were intracardially perfused with 40 ml 0.9% saline (Intermountain life sciences, Z1377) followed by 25 ml of 4% PFA. After perfusion, the left sciatic nerve and the right and left gastrocnemius and tibialis anterior muscles were removed, weighed, and stored in 4% PFA. The spinal cord from T12 to L6 was removed and stored in 4% PFA for retrograde analysis.

### Toluidine blue staining

2 mm of the distal most portions of the branches in the grafts were cut via scalpel for toluidine blue staining. Toluidine blue staining was performed for each branch according to the previously described protocol (Ghnenis et al., 2018). Each distal graft branch was put in 2% osmium tetroxide (Sigma-Aldrich, 75632) diluted 1:1 in Trump’s fixative for 2 h. Nerve segments were then removed and placed in 1× PBS for 10 min to wash remaining osmium and Trump’s. Nerve sections then underwent dehydration using 30, 60, 90, and 100% acetone while the epoxy embedding medium (Sigma-Aldrich, 45359) was prepared. After the dehydration process, the nerves were acclimated to the epoxy using a 1:1 100% acetone:epoxy mixture for 30 min followed by a 1:2 100% acetone:epoxy mixture for another 30 min. Segments were then placed in molds (VWR, cat. no. 103302-482) and submerged in epoxy in a 60 *^o^*C oven overnight. Once polymerized an ultramicrotome fitted with a glass knife was used to cut the nerve into semithin sections before they were placed on glass slides. 1% toluidine blue (ThermoFisher, 348601000) was prepared and used to stain the sections for 30 seconds before washing. Slides were then cover slipped and prepared for imaging.

### Cryosectioning

Spinal cords were removed from 4% PFA and stored in 30% sucrose solution in PBS (ThermoFisher, J65148.A1) overnight. Spinal cords were cut into smaller sections to allow for cross sectional cutting. Samples from between L3-L5, where the sciatic nerve enters the spinal cord, were embedded in Tissue-Tek^®^ O.C.T. Compound, Sakura^®^ Finetek (VWR, cat. no. 25608–930) and frozen at −20°C. 20 μm thick sections were cut on a cryostat and placed on Superfrost glass slides (VWR, 48311-703). Slides were then cover slipped using Fluoroshield (Sigma-Aldrich, cat. no. F6182-20ML) and allowed to dry at 4°C overnight.

### Imaging and counting

Toluidine blue stained sciatic nerves were imaged using a Zeiss Axio Scan Z.1 which allowed for brightfield images of whole nerves (10× magnification), used for total nerve axon counts and axonal density, and high magnification images (60× magnification), required for axon diameter and G-ratio calculation. Qupath software was used to obtain total nerve axon counts and axonal density and ImageJ was used to obtain average axonal diameter and G-ratio measurements as described (Bankhead et al., 2017; Roballo and Bushman, 2019). Total axon counts were taken by manually marking each identifiable axon. Axonal density was obtained by manually counting each identifiable axon in a 20,000 μm^2^ section of the nerve. Average axonal diameter was obtained by measuring the inner, unmyelinated, x and y diameter of a randomly selected subset of axons. G-ratio was calculated by dividing the axon diameter by the diameter of the axon plus the myelin sheath of a randomly selected subset of axons.

A Zeiss 980 inverted confocal microscope was used to obtain fluorescent images of retrograde labeled spinal cords. Whole spinal cord images were obtained at 10× using the tile function of the microscope. Higher magnification (20×) images were also acquired in the ventral and dorsal horns. Qupath software was used to manually count all labeled neurons in the ventral and dorsal horns of the spinal cords. Labeled neurons were only counted if they were contained in the dorsal or ventral horn. The dorsal and ventral sides of the spinal cord were identified by locating the median fissure and anterior spinal artery as landmarks (Watson et al., 2009; Toossi et al., 2021). Dorsal and ventral horns were separated by identifying the lateral spinal nucleus and drawing lines slightly ventral to that point at approximately the division of the 5 and 6 laminae, according to Watson et al. (2009). Only clearly identifiable neurons outlined in stain were counted (Toossi et al., 2021). The number of dorsal horn labeled neurons were then divided by the number of ventral horn labeled neurons to obtain a ratio of dorsal horn (sensory) neurons to ventral horn (motor) neurons.

### Statistical analysis

Rats were randomly sorted into experimental groups. Experimenters assessing sensorimotor and histological outcomes were blinded to the experimental groups during data collection. IBM SPSS software was used to run all statistical analysis. One-way repeated measures ANOVA with Tukey *post-hoc* analysis was performed for all Catwalk and CMAPs data. Two-way ANOVAs with Tukey *post-hoc* analysis were performed for all toluidine blue data. A Wilcoxon signed rank test was used to compare tibial and peroneal retrograde labeling data. Paired T tests were used to compare muscle wet weights in the left gastrocnemius group to the right gastrocnemius group and the left tibialis anterior to the right tibialis anterior group.

## Results

### Study design

**FIGURE 1:**
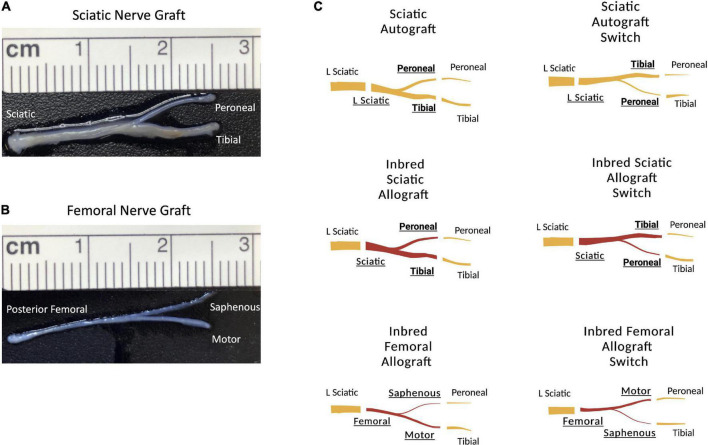
Schematic representation of the surgical groupings and graft types. **(A)** 2.5 cm sciatic nerve graft with peroneal and tibial branches. **(B)** 2.5 cm femoral nerve graft with saphenous and motor branches. **(C)** Schematic representation showing the orientation of the grafts. Original sciatic nerve orientation with the peroneal branch of the graft sutured to the distal peroneal site and the tibial branch of the graft sutured to the distal tibial site. The sciatic switch groups with the opposite, with the peroneal branch of the graft sutured to the distal tibial site and the tibial branch of the graft sutured to the distal peroneal site. Inbred sciatic allografts depicting the same orientations as shown for sciatic autografts. Inbred femoral allografts in the original orientation with the motor branch of the graft sutured to the distal tibial site and the saphenous branch of the graft sutured to the distal peroneal site. In the switched orientation, the motor branch of the graft was sutured to the distal peroneal site and the saphenous branch of the graft was sutured to the distal tibial site.

This study was designed to determine how graft source and orientation influenced regeneration in a branched ablation PNI. 2.5 cm defects of the rat sciatic nerve were created that included the peroneal-tibial bifurcation. The sciatic nerve is a useful model as a result of its mixed sensory and motor nerve morphology in the trunk as well as the peroneal and tibial branches (Irintchev, 2011). For surgical groups, the 2.5 cm defect was bridged with sciatic autografts, inbred sciatic allografts (Figure 1A), or inbred femoral allografts (Figure 1B). Each graft type was tested in two orientations; an original orientation where the tibial and motor branches of sciatic and femoral grafts were sutured to the tibial stump and the peroneal and saphenous were sutured to the peroneal stump; or a switched orientation where the peroneal and saphenous branches in the sciatic and femoral grafts, respectively, were sutured to the tibial stump and the tibial and motor branches of the grafts were sutured to the peroneal stump (Figure 1C). The femoral nerve graft is useful to explore what effect the distinct sensorimotor branch point in this graft would have on regeneration where both the distal branches are mixed (Irintchev, 2011). Both host and donor animals were inbred Lewis rats to minimize immunogenicity as no immunosuppressive treatment was administered.

**FIGURE 2:**
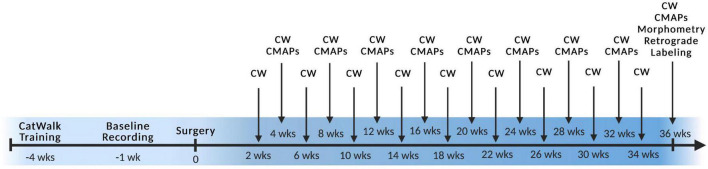
Experimental timeline. Surgery was performed at week 0 with preoperative training and baseline recordings labeled with negative numbers and all postoperative timepoints labeled with positive numbers. CW, Catwalk; CMAPs, compound muscle action potentials. *n* = 5 for sciatic autograft group, *n* = 6 for sciatic autograft, inbred sciatic allografts, and inbred femoral allografts groups.

At the initiation of the study, rats underwent 4 weeks of CatWalk acclimatization and training followed by sciatic nerve surgery with functional and behavioral assessments every 2- or 4-weeks PO (Figure 2). All animals started to show CMAPs and Catwalk recovery by 13 weeks PO. At the study endpoint of 36 weeks, retrograde labeling was performed, muscle wet weight was measured, and nerve cross sections were stained with toluidine blue and analyzed for morphometry of each branch within the graft (Figure 2). Due to the quantity of data gathered, results are shown comparing each graft type by orientation to facilitate analysis and interpretation. Comparisons of the same outcome measures between all groups were also made and can be seen in Supplementary Figures 2–4. Data indicated in the tibial branch represents measures on the branch connected to the tibial stump irrespective of whether the nerve branch within the graft was tibial or peroneal of the sciatic branched grafts or saphenous or motor of the femoral branched grafts. Visa versa for data for the peroneal branch.

### Regeneration by orientation of sciatic autografts

The tibial and peroneal nerves are of significantly different sizes (Figure 1A) and switching the orientation of these branches causes incongruity of graft diameter during surgical coaptation. As graft diameter and autograft harvesting are common surgical considerations in treatment of segmental nerve injuries, experiments sought to determine if graft orientation (i.e., switching the branches) affected outcomes in the 2.5 cm branched ablation model.

**FIGURE 3:**
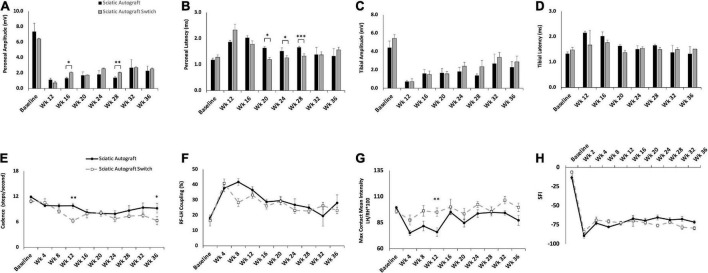
Compound muscle action potentials (CMAP) and gait analysis of the sciatic autograft and sciatic autograft switch groups. **(A)** Amplitude recordings of the peroneal branch of the sciatic nerve. **(B)** Latency recordings of the peroneal branch of the sciatic nerve. **(C)** Amplitudes recorded from the tibial branch of the sciatic nerve. **(D)** Latencies of the tibial branch of the sciatic nerve. *p < 0.05, **p < 0.01, ***p < 0.001 by repeated measures ANOVA with Tukey *post-hoc* test. **(E)** Cadence in steps per second as calculated by the CatWalk software. **(F)** RF-LH coupling showing the percentage of time the left hind (LH) paw preceded the right front (RF) paw in the step cycle. **(G)** Measure of the maximum intensity at the point of maximum contact for the left hind paw/right hind paw*100. **(H)** Sciatic functional index (SFI) recordings as calculated by the CatWalk software. *n* = 5 for sciatic autograft, *n* = 6 for sciatic autograft switch, *p < 0.05, **p < 0.01 by repeated measures ANOVA with Tukey *post-hoc* test. Error bars are standard error of the mean (SEM).

Compound muscle action potential data showed that amplitudes increased in the peroneal branch of the sciatic nerve in the switched orientation (2.03 ± 0.314 mV, 2.03 ± 0.206 mV) at many time points, significantly so at 16- and 28- weeks PO (*p* = 0.03, *p* = 0.004), respectively (Figure 3A). Latencies for the peroneal branch were also decreased in the autograft switch group (1.19 ± 0.072 ms, 1.26 ± 0.0879 ms, 1.32 ± 0.0891 ms) at many time points, significantly at 20-, 24- and 28-weeks PO, respectively (*p* = 0.011, *p* = 0.024, *p* ≤ 0.001) (Figure 3B). Peroneal CMAP amplitudes suggest that the number of reinnervating fibers to distal musculature was improved at early timpoint when the orientation of the graft was switched compared to the non-switched orientation. Decreased peroneal latency in the switched orientation suggests a more rapid conduction speed at these time points, closer to the conduction speed at baseline. Conversely, CMAP amplitudes and latencies for the tibial branch were equivalent at all time points in the autograft group compared to the switched orientation (Figures 3C,D).

CatWalk was performed to assess functional reinnervation as well as behavioral recovery of gait following injury. Catwalk recordings in the sciatic autograft group showed significantly less disruption to their cadence at 12- and 36-weeks PO (9.84 ± 0.60 steps/s, 9.3 ± 1.13 steps/s) compared to the sciatic autograft switch group at these same time points (6.3 ± 0.46 steps/s, 6.3 ± 0.96 steps/s) (*p* ≤ 0.001, *p* = 0.015), indicating injury accommodations were initially made for the sciatic autograft group to maintain a high cadence (Figure 3E). Right front-left hind (RF-LH) coupling is an indication of inter-paw coordination and in uninjured rats for this study, the LH was placed first, and stayed placed 20% longer during a step sequence, than the RF (Figure 3F). Changes in the RF-LH coupling pattern indicate a disruption to inter-paw coordination that can be caused by a reduction in the number of steps from the LH or that LH is on the platform for a longer portion of the step sequence. Increased RF-LH coupling can be seen in both the sciatic autograft and sciatic autograft switch group 4 weeks PO. By 8 weeks PO the sciatic autograft switch group reduced their RF-LH coupling compared to the sciatic autograft group, which increased their RF-LH coupling further. Both groups steadily reduced their RF-LH coupling following 8 weeks PO indicating steady recovery of inter-paw coordination. Max contact mean intensity is presented as a measure of LH/RH*100 and is a measure of the amount of weight borne on the LH foot relative to an intact (RH) control. The sciatic autograft group had significantly less weight borne on the LH compared to the sciatic autograft switch group at all timepoints after injury, although the difference was only significant at 12 weeks PO (76 ± 4, 95.1 ± 4.01, *p* = 0.001), correlating with the cadence results (Figure 3G). This reduction in weight borne on the LH is one of the accommodations made by the sciatic autograft rats to allow for cadence to remain relatively unchanged. Sciatic functional index (SFI) assesses the functional recovery of the sciatic nerve and its contribution to overall gait (Bozkurt et al., 2008). The sciatic autograft and sciatic autograft switch groups had the same functional SFI recovery despite any gait accommodations made by each group (Figure 3H).

**FIGURE 4:**
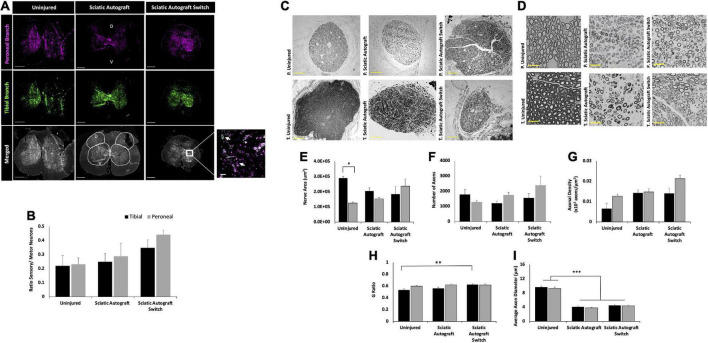
Sciatic autograft and sciatic autograft switch retrograde labeling and nerve morphometry. **(A)** Retrograde labeled spinal cords showing peroneal (magenta) and tibial (green) branch labeling. The dorsal and ventral horns are outlined in the merged image for the sciatic autograft group. Box in the merged image of the sciatic autograft switch column shows a 20× magnification, scale bar is 20 μm. Arrows indicate labeled neurons. Scale bars are 500 μm. **(B)** Ratio of sensory/motor neurons counted from the peroneal (magenta) and tibial (green) nerves. **(C)** Cross section images of the peroneal and tibial nerves taken at 10×. Scale bars are 200 μm. **(D)** 40× images of the toluidine blue stained sections from within the grafts; labeling of images is based on connection of the branch in the graft to the hosts peroneal or tibial distal nerve stump. Scale bars are 20 μm. **(E)** Total cross sectional nerve area taken for the tibial and peroneal branches. **(F)** Total number of axons. **(G)** Axonal density. **(H)** G-ratio. **(I)** Average inner x and y plane axon diameter. *n* = 5 for sciatic autograft, *n* = 6 for sciatic autograft switch, *p < 0.05, **p,0.01, ***p < 0.001 by two-way ANOVA with Tukey *post-hoc* test. Error bars are SEM.

Retrograde labeling was performed to identify any changes in sensory and motor reinnervation through the grafts. Sectioning of the spinal cord showed the distribution of labeled neuronal soma from the peroneal (magenta) and tibial (green) labels (Figure 4A). Localization of sensory (dorsal horn) and motor (ventral) neurons were determined by their location in relation to the median fissure and anterior spinal artery as described previously (Watson et al., 2009; Toossi et al., 2021) (Figure 4A, merged). As expected of mixed sensory and motor nerves, images show an abundance of both magenta and green label in the dorsal and ventral horns for both groups (Figure 4A). A large portion of labeled neurons can be seen in the ventral horn, indicating a majority of tagged neurons were motor neurons. Quantification of the ratio of sensory to motor neurons showed no significant differences between branches or compared to uninjured controls, where the ratio of motor/sensory in both branches was approximately 0.2 (Figure 4B).

Morphological analysis of the nerve size, total axon counts, axonal appearance, and axonal density were performed on toluidine-blue semithin cross sections. Sections were taken within each branch of the graft and the notation in the figures signifies what distal nerve structure the branch in graft was sutured to (i.e., peroneal switch is within the tibial branch of the graft that is connected to the peroneal stump). Figures 4C,D show representative low and high magnification images of the nerve and axons. Nerve area of the tibial branch of uninjured animals was significantly larger than the peroneal branch (2.9 × 10^5^ ± 1.1 × 10^4^ μm^2^ vs. 1.3 × 10^5^ ± 7.2 × 10^3^ μm^2^, *p* = 0.005) which is to be expected based on the visibly noticeable size difference of the tibial and peroneal branches seen in Figure 1A (Figure 4E). This significant difference was not preserved in the branches of the sciatic autograft and sciatic autograft switch groups, but there was a trend for larger nerve area within the tibial portions of grafts irrespective of if the tibial portion of the graft was sutured to the tibial or peroneal nerve. No significant differences were observed for total number of axons and axon density (Figures 4F,G). While not significantly different, the sciatic autograft switch group had a larger nerve area, total number of axons, and axonal density in the peroneal branch than the uninjured or sciatic autograft groups. Considering that the distal peroneal branch was connected to the tibial branch of the graft, where the sample was taken from, it is notable that this was not significant. The G-ratio of the sciatic autograft switch tibial branch (0.627 ± 0.014) was found to be significantly higher than the uninjured tibial branches (0.533 ± 0.021, *p* = 0.007) (Figure 4H). Both tibial and peroneal branches in the uninjured control had significantly larger average axon diameters than both sciatic autograft groups (*p* ≤ 0.001) (Figure 4I). These results indicate that even by 36 weeks PO, that autografts did not replicate the larger axon diameter of uninjured animals but did display G-ratios consistent with their smaller axon diameters.

### Regeneration by orientation of inbred sciatic allografts

**FIGURE 5:**
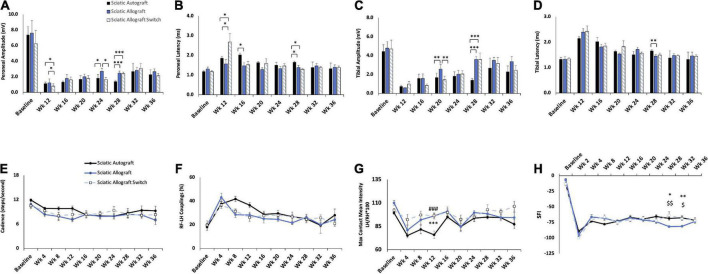
Compound muscle action potential (CMAP) and gait analysis of inbred Lewis to Lewis sciatic allograft and sciatic allograft switch groups. Sciatic autograft is included for reference. **(A)** Amplitude recordings of the peroneal branch of the sciatic nerve. **(B)** Latency recordings of the peroneal branch of the sciatic nerve. **(C)** Amplitudes recorded from the tibial branch of the sciatic nerve. **(D)** Latencies of the tibial branch of the sciatic nerve. *p < 0.05, **p < 0.01, ***p < 0.001. **(E)** Cadence in steps per second as calculated by the CatWalk software. **(F)** RF-LH coupling showing the percentage of time the left hind (LH) paw preceded the right front (RF) paw in the step cycle. **(G)** Measure of the maximum intensity at the point of maximum contact for the left hind paw/right hind paw*100. **(H)** Sciatic functional index (SFI) recordings as calculated by the CatWalk software. *n* = 5 for autograft group and *n* = 6 for inbred sciatic allografts in both orientations. *p < 0.05, **p < 0.01 between sciatic autograft and sciatic allograft, ^###^*p* < 0.001 between sciatic autograft and sciatic allograft switch, ^$^*p* < 0.05, ^$$^*p* < 0.01, ^$$$^*p* < 0.001 between sciatic allograft and sciatic allograft switch. All statistical analysis were repeated measures ANOVA with Tukey post hoc test. Error bars are SEM.

**FIGURE 6:**
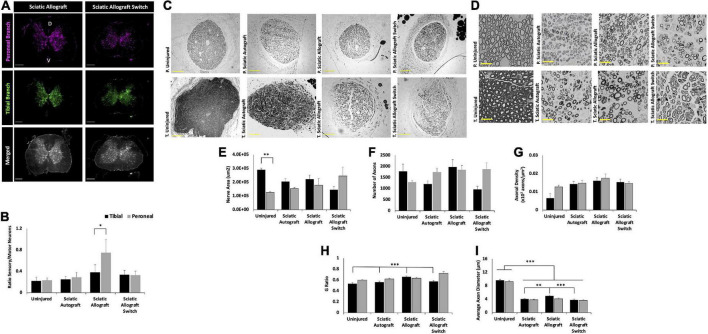
Inbred Lewis to Lewis sciatic allograft and sciatic allograft switch group retrograde labeling and nerve morphometry. Sciatic autograft is included for reference. **(A)** Retrograde labeled spinal cords showing peroneal (magenta) and tibial (green) branch labeling. Scale bars are 500 μm. **(B)** Ratio of sensory/motor neurons counted from the peroneal (magenta) and tibial (green) nerves. *p < 0.05 by Wilcoxon Signed Rank Test comparing tibial and peroneal ratios. **(C)** Cross section images of the peroneal and tibial nerves taken at 10×. Scale bars are 200 μm. **(D)** 40× images of the toluidine blue stained sections from within the grafts; labeling of images is based on connection of the branch in the graft to the hosts peroneal or tibial distal nerve stump. Scale bars are 20 μm. **(E)** Total cross sectional nerve area taken for the tibial and peroneal branches. **(F)** Total number of axons. **(G)** Axonal density. **(H)** G-ratio. **(I)** Average inner x and y plane axon diameter. *n* = 5 for sciatic autograft, n = 6 for sciatic allografts in both orientations, *p < 0.05, **p < 0.01, ***p < 0.001 by two-way ANOVA with Tukey *post-hoc* test. Error bars are SEM.

Evaluation of inbred sciatic allografts was included primarily as an intermediary to the inbred femoral allografts. Sciatic allografts from inbred Lewis rats allows for direct comparison to sciatic autografts to determine the extent that immunogenicity factored into this inbred model. Figures 5, 6 show CMAPs, CatWalk, retrograde labeling and nerve morphometry of the sciatic allograft, sciatic allograft switch and the sciatic autograft groups to facilitate comparison. Peroneal amplitudes and latencies both recovered at a steady state for the sciatic allograft and sciatic allograft switch groups (Figures 5A,B). Some significant differences in peroneal amplitudes were observed at week 12 where sciatic autograft (1.13 ± 0.19 mV, *p* = 0.028) and sciatic allograft (1.22 ± 0.517 mV, *p* = 0.003) were significantly greater than the sciatic allograft switch (0.80 ± 0.333 mV); at week 24 where sciatic allograft (2.71 ± 0.384 mV) was significantly greater than the sciatic autograft (1.83 ± 0.455 mV, *p* = 0.018) and sciatic allograft switch (1.66 ± 0.263 mV, *p* = 0.002); and at week 28 where the sciatic autograft (1.40 ± 0.173 mV) was significantly inferior to sciatic allograft (2.47 ± 0.262 mV, *p* ≤ 0.001) and sciatic allograft switch (2.42 ± 0.184 mV, *p* ≤ 0.001). Peroneal latencies in the sciatic allograft [1.57 ± 0.22 ms (*p* = 0.003), 1.47 ± 0.150 ms (*p* ≤ 0.001), 1.39 ± 0.104 ms (*p* = 0.014)] and sciatic allograft switch groups [2.68 ± 0.429 ms (*p* = 0.028), 1.52 ± 0.173 ms (*p* = 0.003), 1.29 ± 0.043 ms (*p* ≤ 0.001)] showed some differences from sciatic autograft (1.87 ± 0.06 ms, 2.03 ± 0.08 ms, 1.66 ± 0.044 ms) at 12-, 16-, and 28-weeks PO, respectively, but equalized by the last two time points (Figure 5B).

Tibial amplitudes suggest inferior reinnervation in the sciatic autograft (1.69 ± 0.45 mV, 1.40 ± 0.173 mV) compared to sciatic allograft (2.57 ± 0.509 mV, *p* = 0.002) at 20 weeks PO and between the sciatic allograft (3.61 ± 0.295 mv, *p* ≤ 0.001) and sciatic allograft switch groups (3.57 ± 0.692 mV, *p* ≤ 0.001) at 28 weeks PO. The sciatic allograft group also had significantly higher tibial amplitudes than the sciatic allograft switch group (1.44 ± 0.382, *p* = 0.002) at 20 weeks PO before the switch group recovered to higher amplitudes at 24 weeks PO (Figure 5C). Tibial latencies similarly showed some significant differences at 28 weeks PO between the sciatic autograft group (1.66 ± 0.0439 ms) and sciatic allograft group (1.45 ± 0.062 ms, *p* = 0.007) (Figure 5D). Taken together, these results suggest that initially more reinnervation of distal muscle groups occurred for the sciatic allograft group before equalizing at later time points to the reinnervation seen in other groups.

CatWalk measurements of cadence and RF-LH coupling showed equivalent recovery between the sciatic autograft group and both sciatic allograft groups (Figures 5E,F). Max contact mean intensity was significantly higher 12 weeks PO in the sciatic allograft (96.2 ± 3.15, *p* ≤ 0.001) and sciatic allograft switch groups (96.5 ± 2.29, *p* ≤ 0.001) compared to the sciatic autograft group (76 ± 4) (Figure 5G). These patterns are similar to Figure 3 and show that gait accommodations to the injury were unique for the sciatic autograft group. There was no difference in SFI scores between sciatic autograft and allograft groups until 28- and 32-weeks PO when the sciatic allograft group (−81.7 ± 1.39, −81.1 ± 1.44) became significantly worse than the sciatic allograft switch group (−63.5 ± 5.77, *p* = 0.004; −68.7 ± 2.90, *p* = 0.015) and the sciatic autograft group (−68.5 ± 2.98, *p* = 0.02; −67.5 ± 3.22, *p* = 0.004) (Figure 5H). This correlates with the CMAPs data and indicates reinnervation plateaued around 28 weeks PO in the sciatic allograft group.

Retrograde labeling data indicates a retention in mixed nerve morphology in all groups with equivalent ratios of sensory to motor neurons in the uninjured, sciatic autograft and sciatic allograft switch group. However, the sciatic allograft group regenerated significantly more sensory neurons in the peroneal branch (0.75 ± 0.14) than in the tibial branch (0.38 ± 0.25, *p* = 0.034) indicating differences in axonal guidance down each branch (Figures 6A,B). This pattern was not retained in the switch surgery which could indicate a complexity in axonal guidance introduced by the switch surgery.

Representative nerve images for morphometry seen in Figure 6C show the general trend that the tibial branch was larger in the groups that were not switched, while the sciatic allograft group that was switched had a higher nerve area (sections from inside the tibial branch of the graft) in the peroneal branch, as expected (Figure 6E). The total number of axons was larger in the peroneal branch of the sciatic allograft switch group despite having similar axon density in both the peroneal and tibial branches suggesting that the increase nerve area seen in this nerve branch accounted for the increased number of axons when axonal density was calculated (Figure 6F). Representative higher magnification images in Figure 6D show the differences in nerve appearance across groups. G-ratio was significantly higher in the tibial branch of the sciatic allograft group (0.66 ± 0.013) compared to the uninjured (0.533 ± 0.021, *p* ≤ 0.001), sciatic autograft (0.56 ± 0.020, *p* ≤ 0.001), and sciatic allograft switch groups (0.59 ± 0.013, *p* ≤ 0.001) (Figure 6H). The average axonal diameter was also significantly higher in the tibial branch of the sciatic allograft group (4.97 ± 0.233 μm) compared to the autograft (4.07 ± 0.211 μm, *p* = 0.02) and allograft switch group (3.78 ± 0.217 μm, *p* ≤ 0.001), but not compared to the uninjured control (9.64 ± 0.28), which had significantly higher average axon diameter in both branches compared to all groups (*p* ≤ 0.001) (Figure 6I). The tibial branch of the sciatic allograft group showed the best regeneration across groups as indicated by a larger number of axons that had significantly more myelination and axon diameter than other groups.

These results with Lewis to Lewis inbred sciatic allografts, obtained without any immunosuppressive therapy, support that immunogenicity did not play a significantly deleterious role in regeneration in transplants between inbred Lewis rats. The results can be contrasted with our previous findings with nerve allografts from Sprague Dawley donors into Lewis recipients, where the lack of immune suppression significantly decreased regeneration without immune suppression (Santos Roballo et al., 2019). As a basis of comparison, we also conducted a pilot cohort of 2.0 cm branched sciatic autografts and sciatic allografts without any immune suppression, where donor and host animals were outbred Sprague Dawley rats. Significant differences were observed for peroneal and tibial CMAPs at 20 (*p* = 0.017, *p* ≤ 0.001) and 26 weeks (*p* = 0.008, *p* = 0.009) PO, respectively (Supplementary Figures 1A,B). The CMAPs results are corroborated by the total axons counts, which indicate that the total number of axons in the tibial branch (1.7 × 10^3^ ± 196 axons) of the sciatic autograft was significantly larger than the tibial branch (1.1 × 10^3^ ± 153 axons, *p* = 0.012) of the outbred sciatic allograft group (Supplementary Figures 1C,D). The G-ratio in the Sprague Dawley rats was identical across both branches of both groups (Supplementary Figure 1E). This data suggests that immunogenicity is a significant factor for allografting conducted with this outbred strain of Sprague Dawley rats compared to the inbred Lewis rats.

### Regeneration with inbred femoral allografts

Preferential motor reinnervation has been partly attributed to intrinsic factors within the motor branch of the femoral nerve that guide regenerating motor axons into the motor branch (Morita et al., 2008). Femoral allografts were tested in both orientations to determine if this would affect regeneration into the tibial and peroneal branches of the sciatic nerve, which are both mixed sensory and motor (Irintchev, 2011). This was tested by transplanting femoral allografts in the Lewis to Lewis inbred model.

**FIGURE 7:**
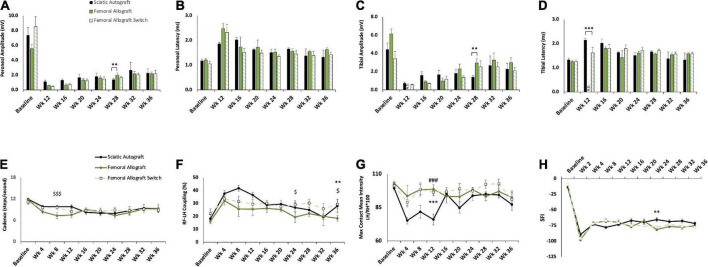
Compound muscle action potential (CMAP) and gait analysis of inbred Lewis to Lewis femoral allograft and femoral allograft switch groups. Sciatic autograft is included for reference. **(A)** Amplitude recordings of the peroneal branch of the sciatic nerve. **(B)** Latency recordings of the peroneal branch of the sciatic nerve. **(C)** Amplitudes recorded from the tibial branch of the sciatic nerve. **(D)** Latencies of the tibial branch of the sciatic nerve. **p < 0.01, ***p < 0.001, N.D. indicates fewer than 3 rats responded. **(E)** Cadence in steps per second as calculated by the CatWalk software. **(F)** RF-LH coupling showing the percentage of time the left hind (LH) paw preceded the right front (RF) paw in the step pattern. **(G)** Measure of the maximum intensity at the point of maximum contact for the left hind paw/right hind paw*100. **(H)** Sciatic functional index (SFI) recordings as calculated by the CatWalk software. *n* = 5 for autograft group and *n* = 6 for inbred femoral allografts in both orientations. **p < 0.01, ***p < 0.001 between sciatic autograft and femoral allograft, ^###^*p* < 0.001 between sciatic autograft and femoral allograft switch, ^$^*p* < 0.05, ^$$$^*p* < 0.001 between femoral allograft and femoral allograft switch. All statistical analysis are a repeated measures ANOVA with Tukey post hoc test. Error bars are SEM.

Femoral allografts showed a plateau in reinnervation by 28 weeks PO that can be seen in CMAPs. CMAPs recording of the peroneal branch show a significantly higher peroneal amplitude in the femoral allograft group (1.98 ± 0.297 mV) compared to the sciatic autograft (1.40 ± 0.173 mV, *p* = 0.009) at 28 weeks PO, but amplitudes in the femoral allograft group plateaued after this point (Figure 7A). Peroneal amplitudes in the femoral allograft switch group also plateaued after 28 weeks PO. Peroneal latency did not differ significantly between groups at any time point but taken with peroneal amplitude, also showed a peak in peroneal branch recovery at 28 weeks PO in the femoral allograft and femoral allograft switch group (Figure 7B). A similar pattern was seen in the tibial branch where tibial amplitude was found to be significantly higher in the sciatic autograft group (1.40 ± 0.173 mV) at 28 weeks PO in comparison to the femoral allograft group (2.96 ± 0.461 mV, *p* = 0.005) with both the femoral allograft and femoral allograft switch group plateauing after this time point (Figure 7C). Tibial latency in the sciatic autograft group was significantly larger in the sciatic autograft (2.15 ± 0.06 ms) compared to the femoral allograft switch group (1.62 ± 0.218 ms, *p* ≤ 0.001) at 12 weeks PO but there were no differences in any group at later time points (Figure 7D). This suggests a threshold of recovery was reached in the femoral allografts regardless of their orientation.

The femoral allograft group showed divergent behavioral recovery at early time points. Cadence was significantly reduced in the femoral allograft group (7.3 ± 0.85 steps/s, *p* = 0.017) at 8 weeks PO compared to the femoral allograft switch group (9.6 ± 0.49 steps/s) (Figure 7E). This normalized to the sciatic autograft and femoral allograft switch groups by 16 weeks PO (Figure 7E). In addition to reduced cadence, the femoral group also had RF-LH coupling percentages that were lower than the other groups but not significantly so (Figure 7F). As the experiment progressed, the femoral allograft group regained a cadence that was in accordance with the other groups, but continued to show a reduced percentage of RF-LH coupling that was significantly lower (19.4 ± 4.13%) than the femoral allograft switch group (29.1 ± 2.95%, *p* = 0.037) at week 24 and was significantly lower (18.5 ± 2.22%) than the femoral allograft switch (29.4 ± 3.38%, *p* = 0.016) and sciatic autograft (28.1 ± 5.24%, *p* = 0.003) at 36 weeks PO.

Mean intensity recordings for the femoral allograft group (98.6 ± 3.58, *p* ≤ 0.001) and femoral allograft switch group (96.5 ± 2.65, *p* ≤ 0.001) were consistent across the duration of the study and were significantly higher than the sciatic autograft group (76 ± 4) 12 weeks PO (Figure 7G). This pattern is unique because it implies that the femoral allograft group were able to maintain their RF-LH coordination and relative weight borne on the LH, but this had to be compensated for by reducing their cadence. SFI measurements were also consistent across group for a large portion of the study, with the femoral allograft group (–81.2 ± 2.04) being significantly different from the sciatic autograft group (–65.4 ± 3.20, *p* = 0.004) only at week 24 before increasing to be in line with the sciatic autograft and femoral allograft switch group at 28 weeks PO (Figure 7H). CatWalk data support CMAPs data showing that functional reinnervation reached completion by 28 weeks in both femoral allograft groups. Additionally, the femoral allograft group made significant gait accommodations after injury that did not resolve as the study progressed.

**FIGURE 8:**
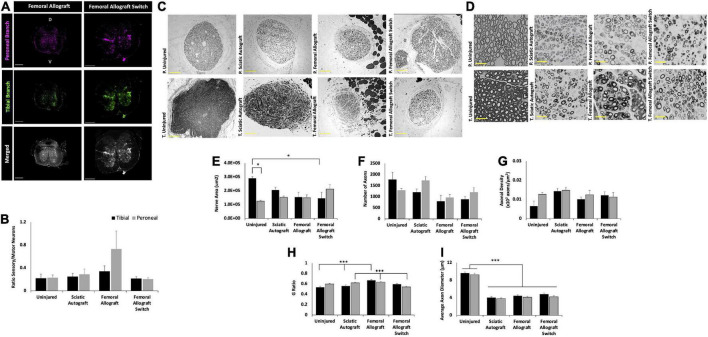
Inbred Lewis to Lewis femoral allograft and femoral allograft switch group retrograde labeling and nerve morphometry. Sciatic autograft is included for reference. **(A)** Retrograde labeled spinal cords showing peroneal (magenta) and tibial (green) branch labeling. Scale bars 500 μm. **(B)** Ratio of sensory/motor neurons counted from the peroneal (magenta) and tibial (green) nerves. **(C)** Cross section images of the peroneal and tibial nerves taken at 10×. Scale bars are 200 μm. **(D)** 40× images of the toluidine blue stained sections from within the grafts; labeling of images is based on connection of the branch in the graft to the hosts peroneal or tibial distal nerve stump. Scale bars are 20 μm. **(E)** Total cross sectional nerve area taken for the tibial and peroneal branches. **(F)** Total number of axons. **(G)** Axonal density. **(H)** G-ratio. **(I)** Average inner x and y plane axon diameter. *n* = 5 for sciatic autograft, *n* = 6 for femoral allografts in both orientations, *p < 0.05, ***p < 0.001 by two-way ANOVA with Tukey *post-hoc* test. Error bars are SEM.

While not significant, spinal cord images of the femoral allograft group show more sensory axons in the peroneal branch compared to the tibial branch, which is consistent with the pattern seen in the sciatic allograft group (Figure 8A). The femoral allograft switch group had equal ratios of sensory/motor neurons in the tibial and peroneal branch (Figure 8B). This data indicates that while reinnervation was largely mixed sensory and motor axons, there could be some indications that graft origin impacts axonal guidance in branched injuries if placed in a nerve of complementary size.

The difference in cross sectional area can be seen in the representative images in Figures 8C,D. The greatest differences in total cross sectional nerve area were between the tibial branch of the uninjured control and the femoral allograft switch group, where nerves were taken from the saphenous graft attached to the distal tibial branch (Figure 8E). While not significant, this pattern was also seen in the reduced number of axons in the femoral allograft switch group (844 ± 136 axons) compared to the uninjured control (1777 ± 326 axons) and indicates that the significantly smaller nerve area in the saphenous branch compared to the tibial branch cannot accommodate as many axons (Figure 8F). However, despite the total area and axonal count differences seen in the saphenous branch, there are not any differences in the axonal density between groups (Figure 8G). Myelin thickness was significantly greater in the tibial branch of the femoral allograft group (0.670 ± 0.013) compared to the sciatic autograft (0.560 ± 0.020, *p* = 0.001) and uninjured controls (0.533 ± 0.021, *p* ≤ 0.001). This is consistent with the results seen in the sciatic allograft tibial branch and indicates more robustly myelinated axons in grafts attached to the distal tibial stump regardless of the graft origin. G-ratio in the peroneal branch was significantly smaller in the femoral allograft switch group (0.6335 ± 0.012) compared to the sciatic autograft (0.620 ± 0.013, *p* = 0.022) and femoral allograft groups (0.542 ± 0.015, *p* = 0.01) indicating that it is not the motor branch of the femoral nerve that is responsible for greater myelination in grafts attached to the distal tibial branch (Figure 8H). As expected with no nerve injury, the uninjured control retained significantly greater average axon diameter in both branches compared to the experimental treatment groups while there were no differences between both of the femoral allograft or sciatic autograft groups (*p* ≤ 0.001) (Figure 8I).

### Comparison across all groups

The same data presented in Figures 3–8 is shown for all groups by each outcome in Supplementary Figures 2–4 to facilitate direct comparison across all groups. CMAP data indicated that the femoral allograft group showed lower amplitudes than the sciatic allograft group at multiple time points across peroneal amplitude, peroneal latency, and tibial amplitude (Supplementary Figures 2A–C). The peroneal amplitude was larger in the sciatic allograft group compared to the femoral allograft group at all time points, significantly so at 16-, and 24-weeks PO (*p* = 0.002, and *p* = 0.001), respectively. The peroneal latency was initially significantly larger (*p* = 0.049) in the femoral allograft group compared to the sciatic allograft group before equalizing at 28 weeks PO. Tibial amplitude was less in the femoral allograft group compared to the sciatic allograft group at all time points, significantly so at 20 weeks PO (*p* ≤ 0.001). Tibial latency was larger in the femoral allograft group at multiple time points, but not significantly so. The significance found across the CMAPs recordings indicates a greater level of reinnervation in the sciatic allograft group in both the peroneal and tibial branches.

CMAP trends between switched orientations were also present at multiple time points. The peroneal amplitude in the sciatic autograft switch group was larger than the femoral allograft switch group at all time points, significantly so at 16- and 24-weeks PO (*p* ≤ 0.001, *p* ≤ 0.001) Peroneal latencies showed more fluctuation over time and were only smaller than femoral allograft switch groups from 20 to 28 weeks PO. Tibial amplitudes were larger in the autograft switch group at a majority of time points, significantly so at 16- and 24-weeks PO (*p* = 0.001, *p* = 0.021). Tibial latencies were less in the autograft switch group compared to the femoral allograft switch group at all later time points, significantly so at 28 weeks PO (*p* ≤ 0.001). These trends indicate greater recovery in the sciatic autograft switch group compared to the femoral allograft switch group. While recovery in the distal peroneal branch of the femoral allograft switch group was consistent across time points, peroneal latencies and tibial amplitude and latencies indicate poor reinnervation to the dorsal muscles of the feet. Recovery in the peroneal and tibial branch of the autograft switch showed greater indications of reinnervation to the dorsal muscles of the feet through larger magnitude amplitudes and smaller latencies.

Additionally, CMAPs taken from the sciatic allograft switch group had larger peroneal and tibial amplitudes at all time points compared to femoral allograft switch group, but only significantly so at 28 weeks PO (*p* ≤ 0.001) in the peroneal branch. Tibial and peroneal latencies were high at early time points in the sciatic allograft switch group before showing improvement and reducing to be equivalent to the femoral allograft switch group, which plateaued at week 16 in each branch. These results, taken in combination with the sciatic autograft switch group could indicate that an incongruous nerve alignment in congruent graft tissue allows for greater reinnervation of distal muscles compared to grafts that are not congruent with the original injury.

When comparing differences for CatWalk across all groups, cadence was the only measure that showed significant differences outside of what was previously discussed. Cadence was significantly larger in the sciatic allograft switch (8.35 ± 0.92 steps/s) compared to the sciatic autograft switch (6.27 ± 0.46 steps/s, *p* = 0.033) 12 weeks PO (Supplementary Figure 3A). The cadence in the femoral allograft switch (8.88 ± 0.64 steps/second, *p* = 0.017) and sciatic allograft switch groups (9.35 ± 0.93 steps/s, *p* = 0.005) were significantly higher than the autograft switch group (6.76 ± 0.70 steps/s) 24 weeks PO. The sciatic autograft switch group had a lower cadence at multiple time points compared to multiple groups, which could point to reduced functional motor reinnervation, and thus reduced movement ability, with the switched autograft nerves at early time points.

Significant comparisons can also be made between groups when looking at nerve morphometry, specifically axonal density. The peroneal branch in the sciatic autograft switch group (0.014 ± 0.003 axons/μm^2^) had a significantly higher axonal density than the femoral allograft switch group (0.012 ± 0.002 axons/μm^2^, *p* = 0.015) which is also seen represented in the higher peroneal amplitudes at week 36 in the sciatic autograft switch group (Supplementary Figure 4C). This correlates with the CMAPs data and suggests that a greater number of axons reinnervated the peroneal branch of the sciatic autograft switch group compared to the femoral allograft switch group. No other significant differences were found between groups.

In addition, the sciatic allograft switch group (0.643 ± 0.018, *p* = 0.001) had a significantly larger G-ratio in the peroneal branch compared to the femoral allograft switch group (0.542 ± 0.015) indicating a more robust recovery of myelination backed up by functional tests (Supplementary Figure 4D). G-ratio was also found to be significantly smaller in the tibial branch of the femoral allograft switch group (0.595 ± 0.013) compared to the sciatic allograft group (0.660 ± 0.013, *p* = 0.002) while the femoral allograft group (0.670 ± 0.013) G-ratio was significantly higher compared to the sciatic allograft switch group (0.574 ± 0.019, *p* = 0.012). This data indicated reduced myelination in switched nerve branches with smaller graft branches compared to larger original orientation branches. Average axonal diameter was significantly higher in the peroneal branch of the sciatic autograft switch group (4.37 ± 0.19 μm) compared to the sciatic allograft switch group (3.65 ± 0.23 μm, *p* = 0.018) which is also in line with CMAPs recordings which show more robust recovery with larger axons in autograft switch groups (Supplementary Figure 4E).

Muscle wet weight measurements compared between groups show no significant muscle loss in either the left gastrocnemius or left tibialis anterior muscles (Supplementary Figure 5). As expected, the right-side gastrocnemius and tibialis anterior were significantly larger than the left (injured) side for all groups showing muscle loss as a result of the injury. The amount of muscle lost was consistent with allografts done in previous reports, indicating there was motor reinnervation that occurred (Roballo and Bushman, 2019).

## Discussion

Previous studies of PNIs have focused on regeneration following lacerations and linear segmental nerve defects, where there is relatively little known about regeneration of segmental nerve defects that ablate entire branch points. Regeneration following ablation of branch points presents a complex scenario for clinicians. The present study was designed to investigate how the orientation and source of branched nerve grafts affects outcomes following an ablation of the branch point where both distal branches are mixed motor and sensory. 2.5 cm ablations of the sciatic nerve in rats, that included the peroneal and tibial branch point (Figure 1A), were bridged with 2.5 cm branched sciatic nerve autografts, inbred sciatic nerve allografts, and inbred femoral nerve allografts. Each type of branched graft was tested in two orientations, with each branch sutured to the distal stump of either the peroneal or tibial nerve of the host animal (Figure 1B). Outcome measures included behavioral, functional, electrophysiological, immunohistochemical and morphometric measures out to 36 weeks.

The primary advancement from this study is that graft source and orientation did not have broad significant effects on outcomes in the long term and experimental end point of 36 weeks. CMAP amplitudes for both sciatic autograft groups were equivalent for both orientations measured from muscles within the foot innervated by the tibial and peroneal nerves (Figures 3A,C). This was also the case for sciatic and femoral allografts in each orientation (Figures 5A,C, 7A,C). CMAP amplitudes recovered to similar extents across all graft types and orientations (Supplementary Figures 2A,C). Nerve morphometry revealed no significant differences in numbers of total axons or axonal density within each branch in either orientation at 36 weeks (Supplementary Figures 4B,C). CatWalk behavioral assessments of max contact mean intensity and SFI, the main assessments used to determine functional recovery of the injury, were equivalent for graft type and orientation (Supplementary Figures 3C,D). There were end point differences in RF-LH coupling and cadence at 36 weeks. However, these are assessments of gait that can indicate injury accommodation based on coordination and speed and may not be direct indications of functional recovery compared to baseline (Deumens et al., 2007; Parkkinen et al., 2013). Therefore, this data collectively indicates that long-term indications of recovery for a 2.5 cm defect of the sciatic nerve that ablated the peroneal/tibial branch point did not show significant differences across multiple outcomes by 36 weeks PO if the defect was bridged with sciatic autograft, sciatic allograft, or femoral allograft in either orientation. This suggests that axon regeneration is robust through branched nerve grafts over the long-term provided that the grafts have matching branching anatomy, somewhat irrespective of how closely matched the diameters of the graft are to the injured nerves.

A secondary advancement of this study was that electrophysiological and behavioral outcome measures did show differences at earlier time points based on inbred graft source and orientation, despite that the differences had largely equalized by the study endpoint. It must be noted that this study’s end point of 36 weeks is longer than most other studies in rat sciatic nerve, typically ranging from 12 to 20 weeks (Deleonibus et al., 2021). CMAP amplitudes for sciatic autograft switch were significantly higher than the original orientation in the peroneal branch at 16 and 28 weeks, with similar (non-significant) trends at 24 weeks. Peroneal latencies were likewise improved in the switched orientation, indicating that regeneration to tissues innervated by the peroneal branch were enhanced when connected to the tibial branch of the graft (Figure 3D). In comparing all groups together, peroneal, and tibial CMAPs for sciatic autografts and allografts in either orientation were significantly superior to femoral allografts at several earlier time points (Supplementary Figure 2).

CatWalk parameters were also affected in all groups at early time points. The cadence was significantly higher for the sciatic autograft group compared to the sciatic autograft switch group at 8 weeks PO and femoral allograft group at 12 weeks PO (Figures 3E, 7E). Additionally, RF-LH couplings showed that there were differences in RF-LH coordination in the sciatic autograft group compared to all groups at early time points (Figures 3F, 5F, 7F). Max contact mean intensity is a measure of weight borne on a foot at the time of maximum contact with the CatWalk platform. It’s worth noting that max contact mean intensity has been used as an indicator of pain in previous studies of diabetic neuropathy, and much smaller sciatic crush/transection injuries, but has not been confirmed as an indicator of pain in large transection studies such as this study (Vrinten and Hamers, 2003; Deumens et al., 2007; Vieira et al., 2020). Max contact mean intensity is here used as a measure of weight borne on the foot as an indicator of LH function. Lesser intensity equates to less weight borne on the LH compared to the RH. The sciatic autograft group showed the largest fluctuation in weight borne on their LH foot which, in addition with cadence and RF-LH coupling indicates a pattern of injury accommodation in which their LH foot was used sparingly and only as an anchor to allow for balance and speed of crossing (Figure 3G). Other experimental groups showed a different pattern of injury accommodation in which the LH initially appeared to be used only for balance, which slowed down their overall cadence. As the nerve regenerated, and cadence improved, the RF-LH coordination improved, and more weight was able to be placed on the LH. Functional recovery of the sciatic nerve, through measurement of SFI, only started to change between groups at 24 weeks PO when the femoral allograft was significantly worse than the sciatic autograft and at 28- and 32-weeks PO where the sciatic allograft was significantly reduced. These SFI changes correlate with significant differences in tibial CMAPs data and indicate a time of significant remodeling to motor innervation that equalized by 36 weeks.

Data from the femoral nerve allografts was of particular interest as it diverged from the sciatic grafts in several ways. The femoral nerve branch is such that the cutaneous branch is exclusively sensory while the motor branch innervates the quadriceps muscle and lacks sensory fibers, whereas both the peroneal and tibial branches of the sciatic nerve contain both sensory and motor neurons (Irintchev, 2011). Preferential motor reinnervation at sensorimotor branch points was established in the femoral nerve and found to be partially mediated by carbohydrate epitopes expressed on motor-associated Schwann cells (Löw et al., 1994; Martini et al., 1994; Morita et al., 2008). It was therefore a possibility that regeneration of motor fibers would be enhanced down the motor branch of the femoral allograft irrespective of whether it was sutured to the tibial or peroneal stump.

Motor reinnervation in the femoral allograft group appears to be different based on branch and time point in this study. While CMAP tibial amplitudes and latencies were improved compared to the peroneal branch after 24 weeks PO in the original orientation femoral allograft group, there were only two rats at 12 weeks PO that responded to tibial stimulation compared to the peroneal branch where all rats responded (Figure 7). This suggests the possibility of early motor reinnervation occurring down the peroneal branch, followed by a later wave of additional motor axons down the tibial branch from 16 to 24 weeks PO. CatWalk recordings support this observation; there was very little disruption to RF-LH coordination and weight bearing on the LH at all time points, but cadence was significantly reduced around 8 weeks PO and SFI was reduced at 24 weeks PO. This could be caused by peroneal motor innervation, which controls dorsiflexion of the foot, occurring early and allowing the animal to coordinate LH stepping and weight bearing with a transient reduction in cadence. Late tibial reinnervation or pruning of incorrectly innervated motor axons accounts for the changes in SFI, which requires innervation of both branches of the sciatic nerve to impact toe spread. An interesting follow up study might use a similar branched grafting procedure but into a femoral nerve branched defect rather than the sciatic to determine if end-organ innervation can still drive preferential motor reinnervation when using mixed branched grafts. We are not aware of any such studies.

Potential reasons that outcomes at earlier time points differed by graft and orientation are not certain but may include factors related to the grafts as well as the defect site. Within this study, where the defect site was standard for all groups, it appears that size disparity in the branches within the graft correlates with earlier differences in outcomes. For example, sciatic autografts show lower peroneal CMAP amplitudes at the earlier time points when the peroneal branch within the graft was sutured to the peroneal stump compared to the switched orientation when the tibial branch within the graft was sutured to the peroneal stump (Figure 3A). The tibial branch in the graft is larger than the peroneal branch (Figure 1A), potentially indicating that axon regeneration at early time points may have facilitated more axons entering the larger (tibial) branch within the graft. Similar trends were observed comparing sciatic allograft to sciatic allograft switch (Figure 5A) and femoral allograft to femoral allograft switch (Figure 7C), where the motor branch within the femoral is larger than the saphenous branch (Figure 1B). Early differences in regeneration may become less evident over time as additional regeneration, remodeling and collateralization occur. As discussed in more detail below, cross sectional area of nerve branches and axon number within the grafts appears to have altered depending on which distal branch it was connected to. Conducting morphometry on nerves at earlier time points when CMAPs and CatWalk differed by graft source and orientation would be beneficial.

A third advancement of this is some of the intriguing findings of nerve morphometry. As stated previously, the cross sections of the tibial branch of the sciatic nerve is larger than the peroneal branch in uninjured animals (1.2 × 10^5^± 7.2 × 10^3^ μm^2^ for peroneal, 2.9 × 10^5^ ± 1.1 × 10^4^ μm^2^ for tibial, *p* = 0.005) (Figure 4E). Thirty-six weeks following treatment with sciatic autografts, the cross-sectional area of the tibial branch within autografts that were sutured to the tibial stump was 2.1 × 10^5^± 2.1 × 10^4^ μm^2^ and the tibial branches with grafts sutured to the peroneal stump (switched orientation) were slightly larger 2.4 × 10^5^± 4.7 × 10^4^ μm^2^, both smaller than in uninjured tibial nerves (Figure 4E). The opposite trend was observed for the smaller peroneal branch, where the cross-sectional area of the peroneal section of the graft sutured to the tibial (1.8 × 10^5^ ± 5.0 × 10^4^) was now larger than in uninjured peroneal (1.5 × 10^5^± 9.2 × 10^3^ μm^2^). This trend was less clear in sciatic and femoral allograft groups, where direct comparison is complicated by donor nerves being obtained from age-matched males that were generally larger than the female recipients (275 g vs. 219 g) and therefore not directly comparable. While differences for autografts did not reach statistical significance, these data suggest that branches within branched autografts may increase or decrease in size depending on what distal nerve branch they are connected to.

Trends in the total number of regenerated axons is also of interest. Axon number in uninjured tibial branches was 1777 ± 326 compared to 1205 ± 140 when the tibial branch of sciatic autografts was sutured to the tibial stump and 2398 ± 588 when the tibial branch within the graft was sutured to the peroneal stump (Figure 4F). Axonal density accordingly increased after injury as the areas of the nerve branches within the autografts became smaller (Figure 4G). Axon number in the tibial branches of sciatic allografts sutured to the tibial distal stump was 1846 ± 200 and was maintained at similarly high numbers, 1875 ± 287 of axons in tibial graft branches sutured to peroneal stumps. The larger motor branch of the femoral nerve similarly attracted regeneration of additional axons when sutured to the peroneal stump (1208 ± 199) compared to when the saphenous nerve in the graft was sutured to the peroneal stump (968 ± 135). We would suggest that future studies on branched ablations should consider using additional animals/group to account for increased variability likely caused by the branching.

A potentially complicating factor in this study is the immunogenicity of the inbred sciatic and inbred femoral allografts as no immunosuppressive therapy was applied. We had previously found that regeneration with both 1 cm and 2.0 cm branched allografts from Sprague Dawley donors into Lewis rats was poor when no immunosuppression was provided (Roballo and Bushman, 2019; Santos Roballo et al., 2019). Results shown in Supplementary Figure 1 indicate that there is still immunogenicity when both donor and host are Sprague Dawley animals, as evidenced by reductions in regeneration in allograft groups. This is likely explained by the outbred nature of the Sprague Dawley strain, having shown immunogenicity in other reports of nerve allotransplantation (Mikesh et al., 2018). Addressing immunogenicity with immunosuppressive therapy is not without complications as validated immunosuppressants tacrolimus and cyclosporin have positive effects on axonal extension and Wallerian degeneration independent of their mechanisms suppressing immune cells (Sunio and Bittner, 1997; Avramut and Achim, 2003). We therefore chose inbred Lewis rats to mitigate immunogenicity of allografts rather than immunosuppressive treatment. Comparison of outcomes for sciatic autografts and inbred sciatic allografts in both orientations show highly comparable outcomes for these groups, suggesting that immunogenicity was not a significant factor for allografts within inbred Lewis rats (Figures 5, 6). The finding that sciatic autografts and inbred allografts show equivalency long-term is meaningful because it allows for direct comparison with the femoral nerve, which has the sensorimotor divisions in branches that is not within the peroneal and tibial branches of the sciatic that both contain sensory and motor neurons.

Retrograde labeling using Alexa Flour conjugated CTb is a method used to trace the motor and sensory neurons in a given nerve and can be used to identify the regeneration nerve morphology (Hirakawa et al., 1992; Zhao et al., 2020; Cui et al., 2022). The retrograde labeling done in this study shows that there were more motor axons compared to sensory axons in all groups, seen in the higher number of labeled neurons in the ventral horn compared to the dorsal horn. When the number of neurons in each horn are counted and compared as a ratio of sensory to motor neurons for each nerve branch, some interesting trends appear. As expected of a nerve with mixed morphology, the uninjured control shows nearly identical ratios of sensory/motor neurons in both the tibial and peroneal branches. This trend is also true for the sciatic autograft group indicating that injury did not meaningfully change the number of sensory and motor neurons. Interestingly, the sciatic allograft switch group also had a nearly identical sensory/motor ratio of neurons while the sciatic allograft group showed significantly more sensory neurons in the peroneal branch compared to the tibial branch. The pattern of reinnervation seen in the sciatic allograft group could possibly support the assertion that sensory axons are more robustly regenerated down the peroneal branch early, followed by motor axons that were diverted to the tibial branch. This pattern was not retained in the switch surgery, which we hypothesize is caused by the added complexity of the size disparity in the graft and distal stump affecting axonal growth cone guidance. The same pattern occurred in the femoral allograft where, while not significantly so, the saphenous branch was innervated by more sensory neurons than the motor branch. Again, when the graft orientation was switched, the pattern did repeat and there were similar ratios of sensory/motor neurons in each branch. This pattern needs to be explored by further study, particularly with more femoral grafts in opposing orientations implanted into the sciatic nerve and femoral nerve to determine if this pattern holds true with more replicates and if it is caused by size disparity in the branches.

SFI is a measure of functional recovery in the sciatic nerve and is calculated using the overall toe spread, the intermediate toe spread and the print length of the injured foot. SFI has yet to be assessed in an injury of the 2.5 cm size and branching the CatWalk. The current study saw initial recovery between 2- and 4-weeks PO before there were only minor fluctuations in recovery. Previous reports using the CatWalk to assess SFI following injury have highlighted limitations of this technology which may impact the SFI scores (Bozkurt et al., 2008). Specifically, Bozkurt et al. (2008), discussed that SFI values can be significantly diminished when rats move with faster speed, which impacts gait (Koopmans et al., 2007). This is a large consideration for this study in which rats moved quickly across the CatWalk platform and achieved what appeared to be relatively little sciatic function according to SFI scores. Additionally, calculating SFI early after injury is challenging and can be inaccurate due to paresis of the injured paw (Monte-Raso et al., 2008). Previous studies that utilize SFI have also found little improvement in later time points following injury in rat models that are much less severe (5 mm nerve gap with direct neurorrhaphy,1 cm autograft, and 5 mm autograft) and complex (linear grafts) than the current study (Shenaq et al., 1989; Lin et al., 2010; Nagao et al., 2011). The static sciatic index, which assesses function while standing still, would be a complementary measure to SFI to better dissect differences in behavioral outcomes.

## Conclusion

This study compared the efficacy with which branched nerve grafts promote repair after ablation of a branch point. Results support that the use of branched grafts is a viable technique for the repair of branched nerve injuries as evidenced by regeneration down individual branches. Long term regeneration is not impacted by the harvest location of the graft or the orientation. This is a promising indication for the use of branched grafts to repair branched nerve defects.

## Data availability statement

The raw data supporting the conclusions of this article will be made available by the authors, without undue reservation.

## Ethics statement

This animal study was reviewed and approved by University of Wyoming IACUC.

## Author contributions

JA helped design the study, performed the surgeries and animals care, collected data, analyzed data, and wrote and edited the manuscript. KR performed some surgeries, training, data acquisition, and edited the manuscript. BS assisted with animal care, acquired CMAPs and CatWalk data, and wrote and edited the manuscript. JB devised experiments, provided supplies and lab space, analyzed data, and wrote and edited the manuscript. All authors contributed to the article and approved the submitted version.
