# Supplementary material for: The effects of graft source and orientation on outcomes after ablation of a branched peripheral nerve
Source: Front Cell Neurosci. 2022 Nov 14;16:1055490. doi: 10.3389/fncel.2022.1055490 (PMC9701849; doi:10.3389/fncel.2022.1055490)
Supplement: Supplementary file 1 [file Presentation_1.PPTX]

## Slide 1
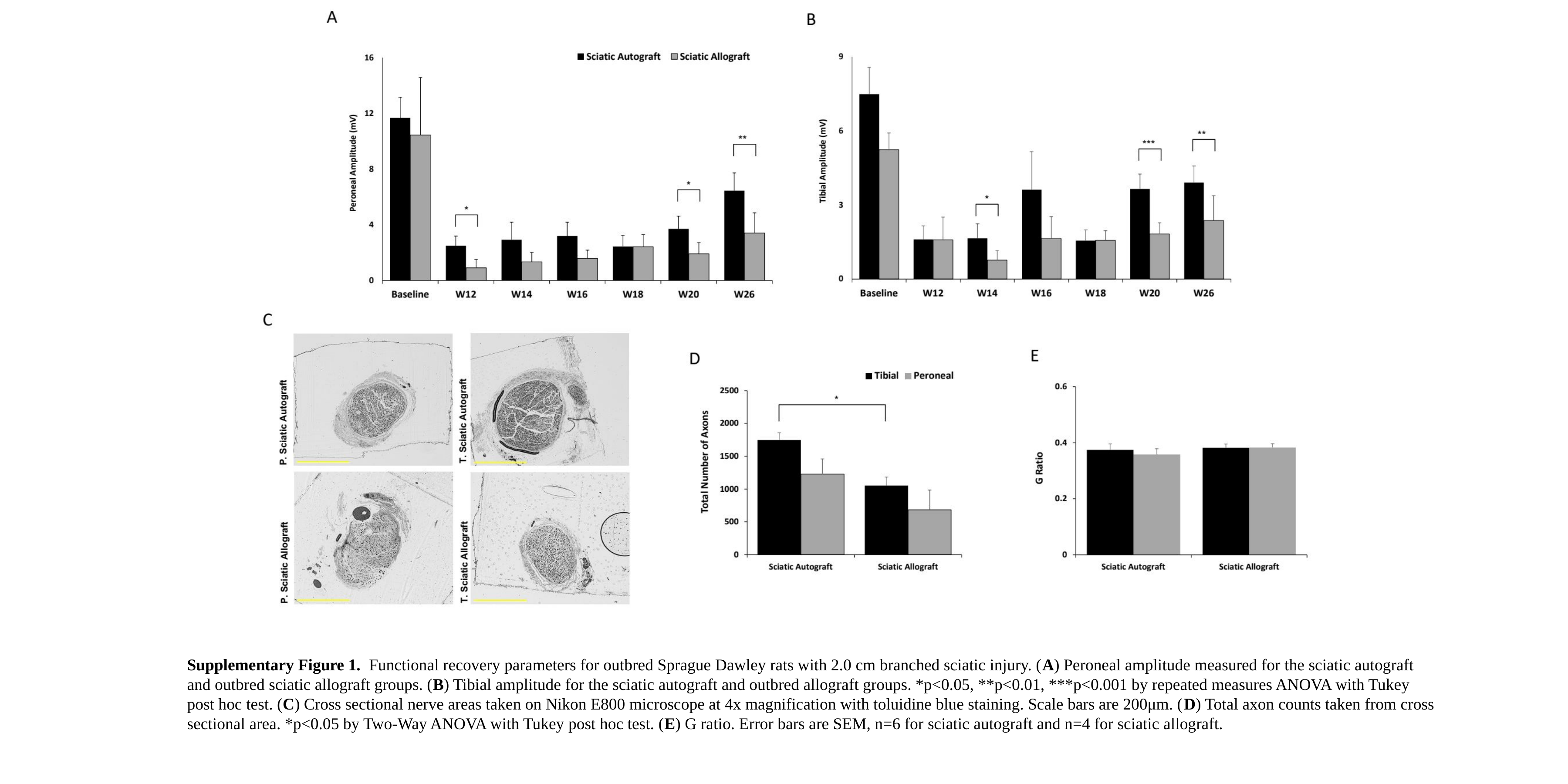

Supplementary Figure 1. Functional recovery parameters for outbred Sprague Dawley rats with 2.0 cm branched sciatic injury. (A) Peroneal amplitude measured for the sciatic autograft and outbred sciatic allograft groups. (B) Tibial amplitude for the sciatic autograft and outbred allograft groups. *p<0.05, **p<0.01, ***p<0.001 by repeated measures ANOVA with Tukey post hoc test. (C) Cross sectional nerve areas taken on Nikon E800 microscope at 4x magnification with toluidine blue staining. Scale bars are 200μm. (D) Total axon counts taken from cross sectional area. *p<0.05 by Two-Way ANOVA with Tukey post hoc test. (E) G ratio. Error bars are SEM, n=6 for sciatic autograft and n=4 for sciatic allograft.

## Slide 2
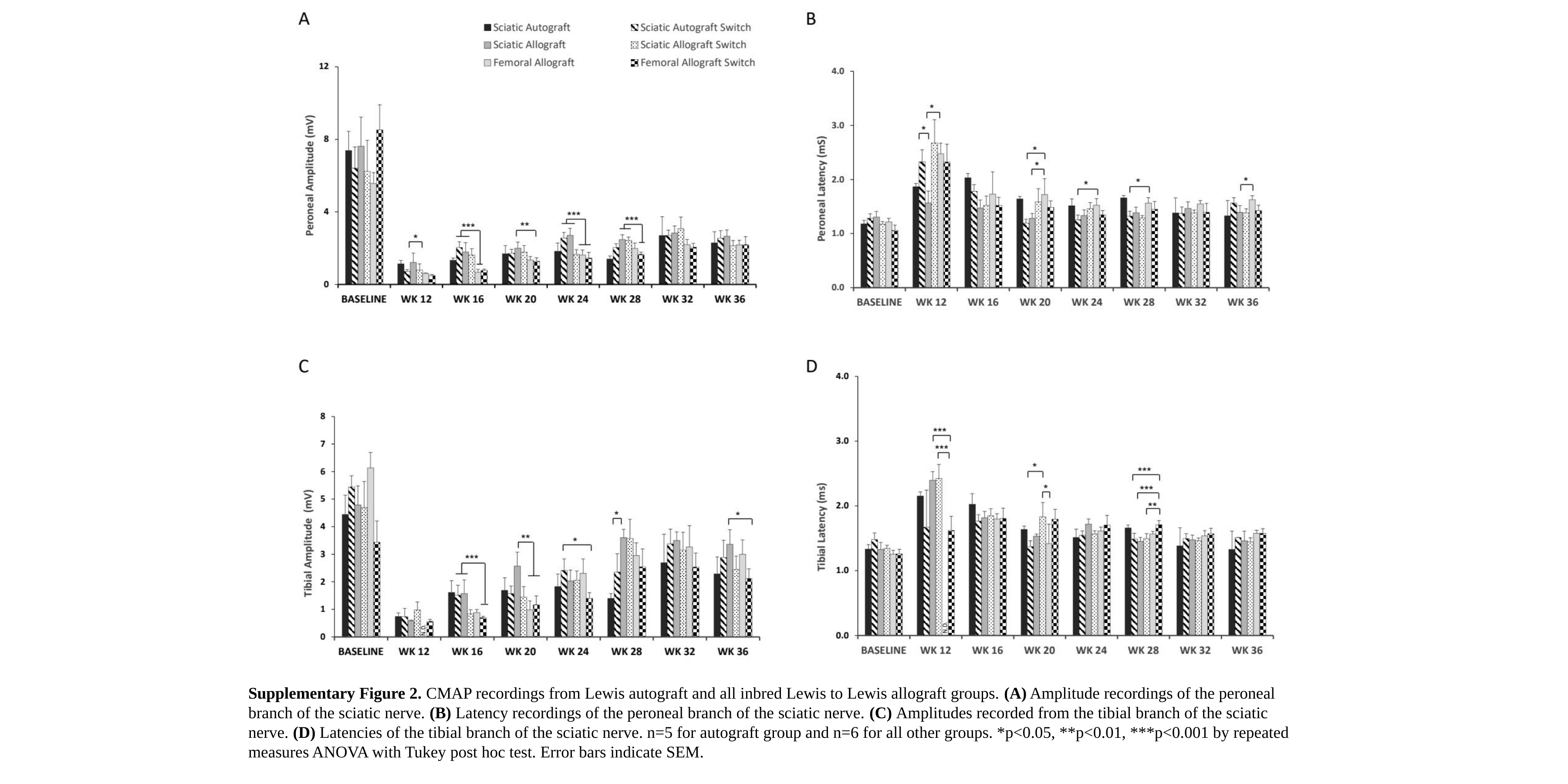

Supplementary Figure 2. CMAP recordings from Lewis autograft and all inbred Lewis to Lewis allograft groups. (A) Amplitude recordings of the peroneal branch of the sciatic nerve. (B) Latency recordings of the peroneal branch of the sciatic nerve. (C) Amplitudes recorded from the tibial branch of the sciatic nerve. (D) Latencies of the tibial branch of the sciatic nerve. n=5 for autograft group and n=6 for all other groups. *p<0.05, **p<0.01, ***p<0.001 by repeated measures ANOVA with Tukey post hoc test. Error bars indicate SEM.

## Slide 3
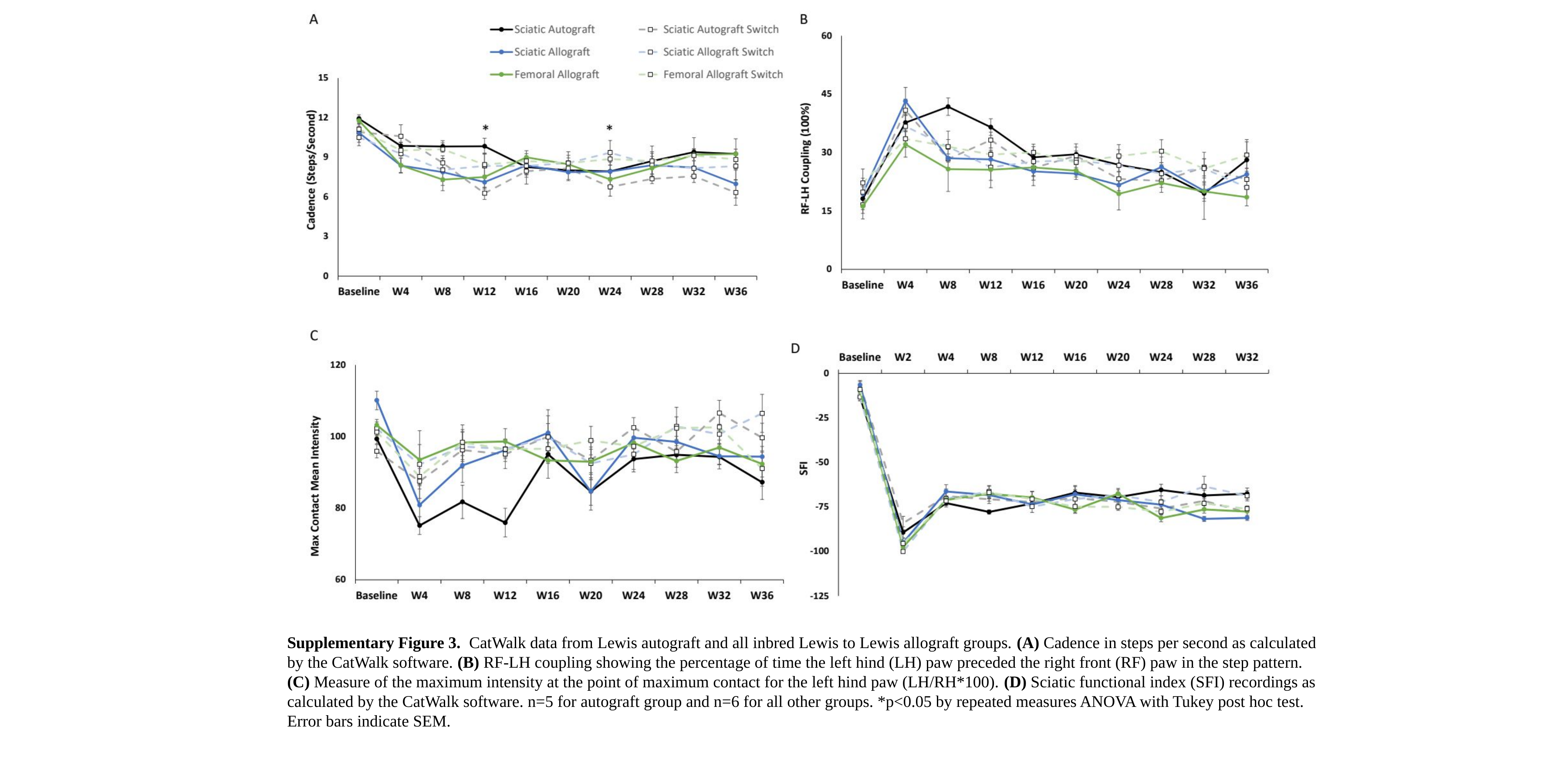

Supplementary Figure 3. CatWalk data from Lewis autograft and all inbred Lewis to Lewis allograft groups. (A) Cadence in steps per second as calculated by the CatWalk software. (B) RF-LH coupling showing the percentage of time the left hind (LH) paw preceded the right front (RF) paw in the step pattern. (C) Measure of the maximum intensity at the point of maximum contact for the left hind paw (LH/RH*100). (D) Sciatic functional index (SFI) recordings as calculated by the CatWalk software. n=5 for autograft group and n=6 for all other groups. *p<0.05 by repeated measures ANOVA with Tukey post hoc test. Error bars indicate SEM.

## Slide 4
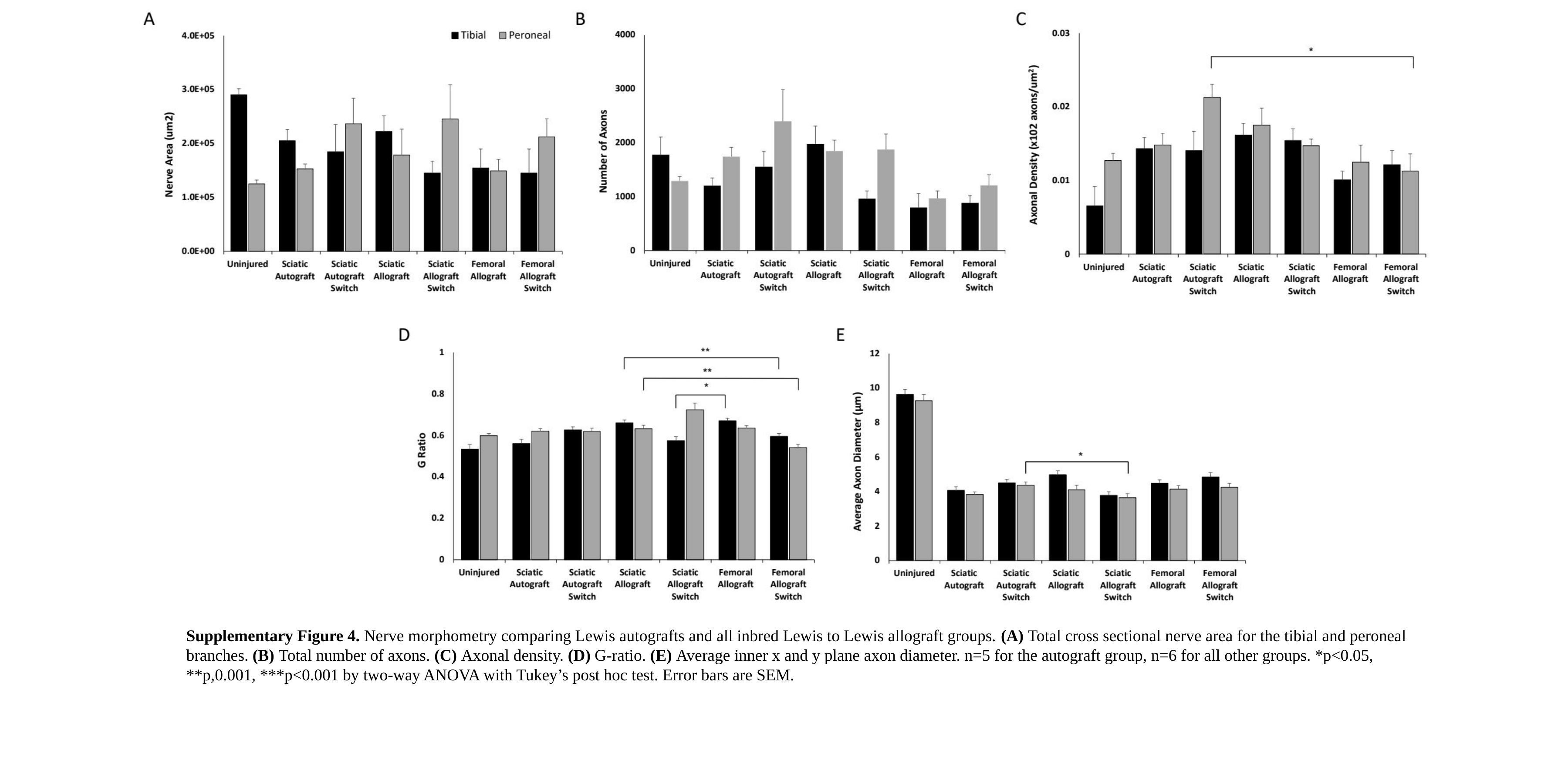

Supplementary Figure 4. Nerve morphometry comparing Lewis autografts and all inbred Lewis to Lewis allograft groups. (A) Total cross sectional nerve area for the tibial and peroneal branches. (B) Total number of axons. (C) Axonal density. (D) G-ratio. (E) Average inner x and y plane axon diameter. n=5 for the autograft group, n=6 for all other groups. *p<0.05, **p,0.001, ***p<0.001 by two-way ANOVA with Tukey’s post hoc test. Error bars are SEM.

## Slide 5
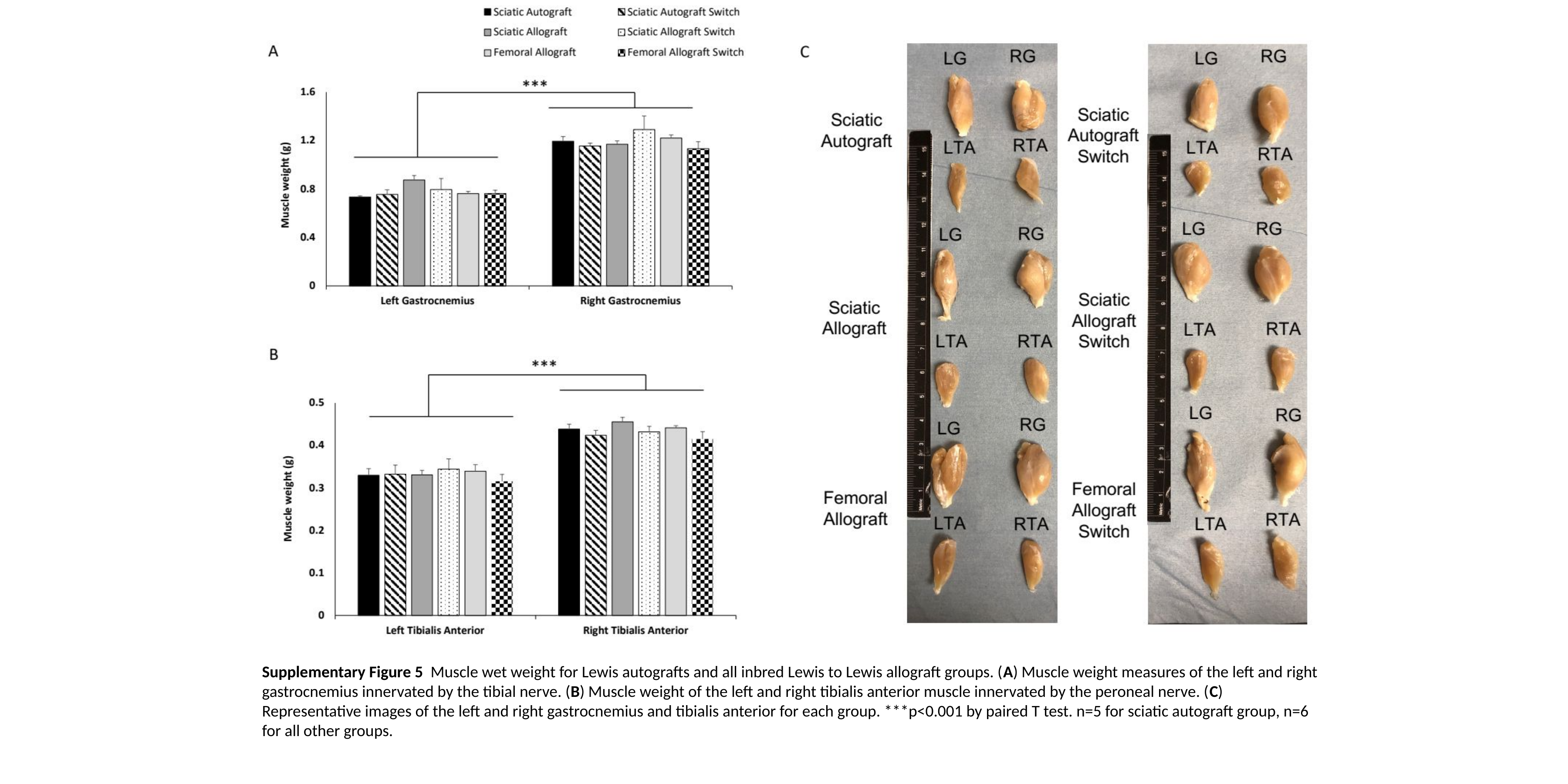

Supplementary Figure 5 Muscle wet weight for Lewis autografts and all inbred Lewis to Lewis allograft groups. (A) Muscle weight measures of the left and right gastrocnemius innervated by the tibial nerve. (B) Muscle weight of the left and right tibialis anterior muscle innervated by the peroneal nerve. (C) Representative images of the left and right gastrocnemius and tibialis anterior for each group. ***p<0.001 by paired T test. n=5 for sciatic autograft group, n=6 for all other groups.
